# Socio-Demographic Determinants, Dietary Patterns, and Nutritional Status Among School-Aged Children in Thulamela Municipality, Limpopo Province, South Africa

**DOI:** 10.3390/children13010065

**Published:** 2025-12-31

**Authors:** Rotondwa Bakali, Vivian Nemaungani, Tshifhiwa Cynthia Mandiwana, Lavhelesani Negondeni, Selekane Ananias Motadi

**Affiliations:** 1 Department of Nutrition, Faculty of Health Sciences, University of Venda, Thohoyandou 0950, South Africa; 22004885@mvula.univen.ac.za (R.B.); 22006604@mvula.univen.ac.za (V.N.); tshifhiwa.mandiwana@univen.ac.za (T.C.M.); 2 Department of Public Health, Faculty of Health Sciences, University of Venda, Thohoyandou 0950, South Africa; lavhelesani.negondeni@univen.ac.za

**Keywords:** socio-demographic factors, food consumption patterns, nutritional status, children, association

## Abstract

**Highlights:**

**What are the main findings?**

• The study uncovered a concerning pattern where many children suffered from growth delays, indicated by stunting, alongside a rising prevalence of overweight conditions and obesity. This demonstrates that both forms of malnutrition are affecting the same population.
• Nutritional outcomes were strongly associated with parental education, marital status, and household income, as well as with healthy eating behaviors such as regular breakfast consumption and the frequent intake of vegetables and dairy products.

**What are the implications of the main findings?**

• Schools are well positioned to implement breakfast programs and promote healthy eating behaviors. Strengthening school nutrition initiatives could help combat both undernutrition and unhealthy weight gain.
• Encouraging frequent intake of nutrient-rich foods like vegetables and dairy products, along with consistent meal patterns, should be a priority in public health nutrition campaigns.

**Abstract:**

**Background:** Childhood undernutrition and overnutrition continue to be major public health challenges in South Africa. There is limited evidence on how socio-economic factors and dietary behaviors influence nutritional outcomes among school-aged children, particularly in rural areas such as Thulamela Municipality. **Objective:** This study aimed to examine the socio-demographic determinants, dietary patterns, and nutritional status among school-aged children in Thulamela Municipality, Limpopo Province, South Africa. **Methods:** A cross-sectional survey was conducted with 347 children aged 8–12 years. Simple random sampling was used to select eight villages from a total of 227 within the municipality. A snowball sampling method was used to recruit eligible children. Data on socio-demographic characteristics, including the child’s sex, parental education level, marital status, and employment status, were collected. Additionally, their dietary habits and meal frequency patterns were collected using structured questionnaires. Anthropometric measurements including height, weight, and BMI-for-age were obtained following WHO growth standards. Associations between variables were assessed using chi-square tests, with *p*-values < 0.05 considered statistically significant. **Results:** The prevalence of severe and moderate stunting was 20.5% and 21.0%, respectively. Overweight conditions and obesity affected 32.6% and 16.2% of participants, respectively. Parental education (*p* = 0.027), marital status (*p* = 0.001), and household income (*p* = 0.043) showed significant associations with height-for-age and BMI-for-age Z-scores. Additionally, regular breakfast consumption and the frequent intake of vegetables and dairy products were positively associated with improved nutritional outcomes (*p* < 0.05). **Conclusions:** The nutritional profile of school-aged children in Thulamela Municipality reflects a double burden of malnutrition, with concurrent high rates of stunting, overweight conditions, and obesity. Interventions that promote balanced diets and address socio-economic disparities are crucial for improving child growth and overall health. Socio-economic factors, including parental education, marital status, and household income, were significantly associated with children’s height-for-age and BMI-for-age. Furthermore, the regular consumption of breakfast, vegetables, and dairy products was associated with better nutritional outcomes, highlighting the influence of both dietary behaviors and socio-demographic determinants on child growth and health. Implementing nutrition education programs within schools that emphasize the value of balanced diets and highlighting the significance of eating breakfast regularly and incorporating vegetables and dairy products into daily meals is important. These programs should include both children and their caregivers to support regular healthy eating behaviors at home and in school. Additionally, schools should carry out regular growth monitoring and nutritional assessments to identify early indications of undernutrition or overnutrition, enabling prompt referrals and interventions for children who may be at risk.

## 1. Introduction

The dietary habits of school-aged children play a crucial role in supporting their physical growth, cognitive development, and academic performance [1]. Despite this, numerous studies have documented that many children continue to exhibit poor dietary practices and face various forms of malnutrition [2,3]. Unhealthy eating behaviors established during childhood have been associated with an increased risk of chronic conditions, including diabetes and obesity, later in life [4]. In many low- and middle-income countries, children’s diets often lack diversity, with limited intake of fruits, vegetables, and other nutrient-dense foods [5]. Consequently, their meals tend to be high in saturated fats, low in dietary fiber, and inadequate in essential micronutrients [6].

Children’s food preferences are frequently influenced by taste rather than nutritional value, which can lead to nutrient deficiencies and impair both physical growth and cognitive development [7,8]. The availability of unhealthy snacks from school tuck shops and nearby food vendors further shapes these choices, highlighting the influence of the surrounding food environment [7]. However, meals provided at home remain a key determinant of children’s dietary intake, as they reflect what is available within the household [4]. Sipple [9] reported that although many children disliked milk, the majority still consumed it regularly because it was a staple at home. Similarly, Viljakainen et al. [10] found that children from households with lower parental education levels were more likely to consume less nutritious foods.

Beyond dietary practices, children’s nutritional status is also shaped by socio-economic and environmental factors [11,12]. Globally, the prevalence of overweight conditions and obesity among children continues to rise, partly due to an increased consumption of processed and energy-dense foods [13,14]. There is a well-documented association between dietary patterns and anthropometric outcomes, with inadequate nutrition negatively impacting not only physical health but also learning potential and school performance [15,16].

In Thulamela Municipality, emerging evidence suggests that poor eating habits and related health concerns are increasingly common among school-aged children [17]. Ligege [2] highlights that many primary school children fail to meet the recommended dietary guidelines, placing them at risk of malnutrition. However, there is limited research examining how socio-demographic factors influence dietary patterns and nutritional status among school-aged children in Thulamela Municipality, Limpopo Province. Understanding these relationships is crucial, as inadequate nutrition during childhood can impair growth and development. This study seeks to address this gap by investigating the socio-demographic determinants, dietary patterns, and nutritional status of school-aged children in the region, providing insights into both undernutrition and overnutrition.

Guided by the 1990 UNICEF Conceptual Framework [18], the study focuses on children aged 8–12 years, exploring how socio-demographic characteristics shape their dietary behaviors and nutritional outcomes. The primary research question was as follows: what are the socio-demographic determinants, dietary patterns, and nutritional status of school-aged children in Thulamela Municipality, Limpopo Province, South Africa?

## 2. Methods

### 2.1. Study Design and Setting

A cross-sectional study design was employed to investigate the socio-demographic characteristics, food consumption patterns, and nutritional status of children aged 8–12 years in Thulamela Municipality. Thulamela is one of the four municipalities located in the Vhembe District of Limpopo Province. The municipality was purposively selected due to the rapid development of shopping malls and food outlets in the area. These commercial developments have increased the accessibility of various food options to the surrounding villages, which may have both positive and negative implications for children’s dietary habits and food consumption patterns.

Data was collected between March and June 2025 by nutrition and biokinetics students from eight villages within the municipality. Prior to data collection, the students received two weeks of intensive training from the research team to ensure consistency and accuracy in the data gathering procedures.

### 2.2. Study Population, Sampling Size, and Sampling Procedure

Thulamela Municipality comprises 227 villages, which vary widely in population and land area. Small villages, such as Shingwidzi (11 people; 0.86 km^2^), Sterkstroom (39; 0.05 km^2^), and Thembaluvhilo (43; 0.09 km^2^), have very low populations and occupy compact areas. Medium-sized villages, including Altein (1759; 2.15 km^2^), Basani (3408; 4.39 km^2^), and Dzingahe (2862; 2.14 km^2^), have populations ranging from about 1000 to 5000 and cover areas between 1 and 5 km^2^. Larger villages and towns, such as Thohoyandou (69,453; 42.62 km^2^), Lwamondo (20,218; 20.72 km^2^), Mukula (8209; 10.41 km^2^), and Itsani (11,473; 6.89 km^2^), are highly populated and occupy more extensive land areas. Some settlements, while moderately populated, cover very large areas, such as Thulamela NU (1399; 2140.39 km^2^) and Kruger National Park (55; 2935.88 km^2^). Overall, the municipality exhibits a mix of densely populated small villages, moderately populated medium villages, and sparsely populated large-area settlements, reflecting both rural and urban characteristics. The total population of the municipality is 618,462, of which 139,529 are children. In terms of language, 64% of the population speak Tshivenda, while 33% speak Xitsonga. A cluster sampling technique was used to select villages for the study. In this method, the 227 villages were first grouped into clusters based on their geographical location and population characteristics to ensure representativeness. Each cluster included villages with similar population sizes and proximities. Eight clusters were then randomly selected using a lottery method, in which each cluster was assigned a unique number, all numbers were placed in a container, and a researcher, blindfolded, drew the numbers randomly. All villages within the selected clusters were included in the study. This approach minimized logistical challenges and ensured that different types of villages were proportionally represented.

At the household level, a snowball sampling method was used to recruit eligible children. To reduce bias from closely connected peer networks, referrals were initiated from multiple starting points across different areas within each selected village. The research assistants initially identified households with at least one child within the target age range of 8–12 years. After completing the interviews and anthropometric assessments in one household, parents or guardians were asked to refer the team to another household with a child in the same age group. This process continued until the desired sample size of 347 participants was achieved.

In cases where more than one eligible child resided in the same household, only one child was included in the study. The anthropometric and dietary data of the first child were recorded, under the assumption that children within the same household consume similar foods.

Children with chronic medical conditions that could influence dietary habits such as those requiring medications that affect appetite or metabolism were excluded. Similarly, children with physical disabilities that limited participation in anthropometric measurements were not included, due to the lack of suitable equipment for accurate height assessment.

The sample size was determined using Slovin’s formula [*n* = *N*/(1 + Ne^2^)] [19], where *N* represents the population of 139,529 children. A 5% margin of error (e = 0.05) and a 95% confidence level were applied, yielding a minimum required sample of 399 participants. To accommodate potential non-responses or withdrawals, an additional 10% was added, resulting in a target sample of 439 children. However, the final sample size was reduced to 347 due to participant withdrawals.

### 2.3. Data Collection and Variables Measured

Data were collected using a structured questionnaire covering socio-demographics, dietary patterns, and a record sheet for anthropometric measurements. To ensure the validity of the questionnaire, a comprehensive review of the relevant literature was conducted, and established questions from similar studies were adapted where appropriate. Additionally, the questionnaire was piloted and pretested to assess clarity, relevance, and consistency, allowing for necessary adjustments before full deployment. To ensure reliability, the questionnaire was then translated into Tshivenda and Xitsonga using forward and back-translation by linguists from the Matshaya Edward Razwimisani (M.E.R) Mathivha Centre for African Languages. To assess reliability, 10% of the questionnaires were randomly re-administered to the same children. Data collection was organized into two stations to ensure efficiency and accuracy. The first station focused on gathering information related to socio-demographic characteristics and food consumption patterns using a structured questionnaire administered to the participants or their caregivers. The second station was dedicated to anthropometric assessments, where the children’s weight and height were measured following standardized procedures.

Anthropometric measurements were conducted following the World Health Organization (WHO)’s standard procedures [20]. Height and weight were recorded twice for each participant using calibrated instruments to ensure accuracy. During measurements, children were barefoot and dressed in light clothing to minimize error. A portable stadiometer was utilized to measure height to the nearest 0.1 cm, while body weight was measured to the nearest 0.01 kg using a portable Seca solar scale (Model 0213; Seca, Hammer Steindamm, Hamburg, Germany). Before each measurement session, both the stadiometer and the scale were calibrated using standard steel tapes and calibration weights to verify precision [21].

The nutritional status of the children was determined using the WHO growth reference standards [22]. Height and weight measurements were converted into age- and sex-specific Z-scores including height-for-age, weight-for-age, and body mass index-for-age using the WHO Anthro and AnthroPlus softwares Version 1.0.4.

### 2.4. Definition of Thinness, Stunting, Overweight Conditions, and Obesity

The children’s anthropometric status was determined using the classification cut-offs recommended by the World Health Organization [22]. Weight and height measurements were converted into age- and sex-specific Z-scores using the WHO Anthro and AnthroPlus softwares, which were then used to categorize each child’s nutritional status [22]. The calculated Z-scores were used to categorize each child’s anthropometric status based on the World Health Organization (WHO) reference standards. Stunting was defined as low height-for-age, where a height-for-age Z-score < −2 SD indicated stunting and a Z-score < −3 SD showed severe stunting. Thinness among school-aged children and adolescents was classified using BMI-for-age, with a Z-score < −2 SD indicating thinness and a Z-score of < −3 SD indicated severe thinness. BAZ ≥ −2 SD and ≤ +1 SD was considered normal. On the upper end of the spectrum, BAZ > +1 SD and ≤ +2 SD classified a child as overweight, while a BAZ > +2 SD indicated obesity.

### 2.5. Dietary Assessment

The dietary assessment was collected using a 24 h dietary recall conducted over four consecutive days using the multiple-pass method, which allowed detailed capture of foods and beverages consumed. Additionally, a food frequency questionnaire (FFQ), adapted from the 2005 National Food Consumption Survey, was used to assess usual dietary intake and overall consumption patterns. The FFQ was translated into Tshivenda and Xitsonga, with back-translation performed to ensure linguistic consistency. This adapted FFQ has been previously validated and applied in South African studies on child nutrition. Dietary data were subsequently quantified and analyzed to evaluate children’s nutritional intake patterns.

All field workers received training on standardized administration techniques to enhance data quality and minimize recall bias. The instrument was pretested and piloted among children with similar demographic and cultural backgrounds to verify clarity, relevance, and appropriateness, and the feedback obtained was used to refine the questionnaire. Visual aids and portion-size guides were also provided to improve estimation accuracy.

The resulting dietary data were analyzed to determine dietary patterns, meal frequency, and dietary diversity, which were subsequently compared with anthropometric indicators to assess the nutritional status of the children.

### 2.6. Ethical Clearance

Ethical approval for this study was obtained from the Research Ethics Committee of the University of Venda (FHS/25/NUT/03/2603). Before the study commenced, approval was obtained from the traditional leaders of the eight selected villages. Consulting with the chiefs ensured community support, enabled access to households, and promoted trust and collaboration between the research team and local residents, which was crucial for conducting the study in an ethical and culturally appropriate manner. All study procedures were conducted in accordance with the Declaration of Helsinki [23], the Good Clinical Practice guidelines, and relevant South African legislation. Prior to participation, both children and their parents or caregivers were provided with detailed oral and written information about the study, including its objectives, procedures, and any potential risks. Written informed consent was obtained from all parents or guardians, and assent was secured from the participating children. For parents or caregivers who were illiterate, the researcher provided a verbal explanation of the informed consent form in Tshivenda and Xitsonga, the local languages spoken in this municipality, and participants who agreed for their children to take part provided a thumbprint as a form of consent.

### 2.7. Statistical Analysis

Multiple measures were taken to ensure the accuracy and reliability of data collection. Completed questionnaires were carefully checked for completeness and consistency, with any unclear responses clarified before entry. Data was systematically coded and entered into Microsoft Excel (version 2016) using a double-entry method to reduce errors. Anthropometric measurements were performed by trained personnel following standardized procedures, with measurement tools calibrated before each session, and classified using WHO reference standards to ensure accuracy. During analysis, SPSS software (version 29; SPSS Inc., Chicago, IL, USA) was used and the dataset was reviewed for missing values, outliers, and inconsistencies. A composite dietary quality score (0–100) was developed from five equally weighted components: healthy food consumption, unhealthy food consumption, meal pattern regularity, food variety, and water intake. Each component was standardized to a 0–20 scale based on available responses. The final score was calculated by averaging valid component scores (minimum 3 components required) and converting to a 0–100 scale. Associations between categorical variables were evaluated using a Pearson chi-square test. An ordinal logistic regression was employed to identify the relationship between dietary habits, demographic variables, and the nutritional status of children. A *p*-value less than 0.05 (*p* < 0.05) was considered statistically significant for both the Pearson chi-square test and the ordinal logistic regression. A one-way ANOVA test was conducted to test for significant differences in dietary quality scores across income categories. Furthermore, a two-sample t-test was used to determine if dietary quality scores differed among male and female children. Moreover, we also computed a multiple linear regression analysis to examine how sex, age, and income predicted the dietary quality score of children.

## 3. Results

The study included a total of 347 participants, of whom 184 (53%) were girls and 163 (47%) were boys. Sex was significantly associated with height-for-age (*p* = 0.012). About 167 (48.1%) of the participants’ parents had tertiary education while 149 (42.9%) had high school education and 17 (4.9%) did not have any formal education. A significant association was observed between parental education and height-for-age (*p* = 0.027). Of the parents of the participants, 159 (45.8%) were single while 123 (35.4%) were married and 35 (10.1%) were cohabiting. A positive association existed between parental marital status and height-for-age (*p* = 0.001) and BMI-for-age (*p* = 0.034). Most, 187 (53.9%), of the parents were unemployed while 160 (46.1%) were employed. No significant association was observed between employment status and nutritional indicators (Table 1).

Most, 199 (57.3%), households had a monthly income of less than ZAR 5000.00 (USD 28.89) while 66 (19%) had an income between ZAR 5000–ZAR 10,000 (USD 288.93–577.86) and 44 (12.7%) had an income between ZAR 10,000–ZAR 20,000 (USD 577.86–1155.72). Monthly household income was positively associated with height-for-age (*p* = 0.008) and BMI-for-age (*p* = 0.043). The majority, 199 (57.3%), of the households spend less than ZAR 2000 (USD 115.57) on food per month. No association was observed between money spent on food and nutritional indicators (Table 2).

**Figure 1:**
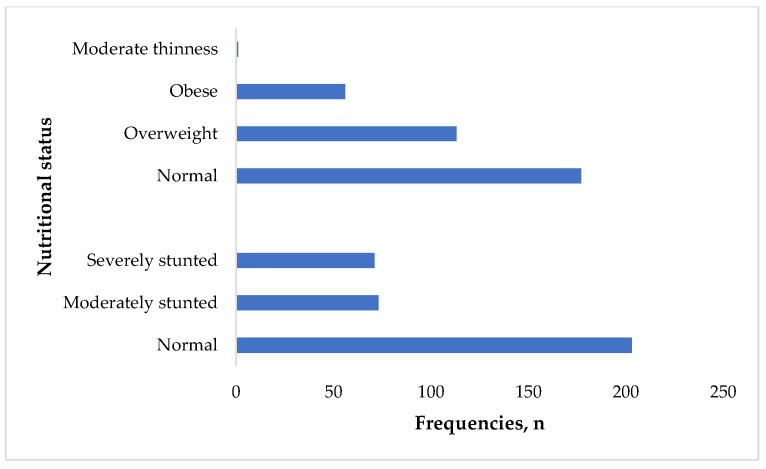
Anthropometric status of the participants (*n* = 347).

The prevalence of moderate and severe stunting was 73 (21%) and 71 (20.5%), respectively. Only 1 (0.3%) of the participants had moderate thinness. About 113 (32.6%) of the participants were overweight while 56 (16.2%) were obese (Figure 1).

Most, 277 (79.8%), of the participants consumed vegetables daily while 74 (21.3%) ate fruits daily and 103 (29.8%) ate fruits and dairy daily. About 130 (37.5%) of the participants had sugary drinks daily while 99 (28.5%) consumed fast food a few times per week. Only 20 (5.8%) had whole grains daily. A significant association was observed between whole grains and height-for-age (*p* = 0.001). Daily consumption of vegetables was associated with height-for-age (*p* = 0.012). Daily consumption of dairy was associated with BMI-for-age (*p* = 0.001). No association was found between fast food consumption, height-for-age, and BMI-for-age (Table 3).

Table 4 illustrates meal frequency and its association with nutritional indicators; breakfast was significantly associated with height-for-age (*p* = 0.001) and BMI-for-age (*p* = 0.051). No association was observed between lunch, dinner, and nutritional indicators.

Table 5 presents the association between dietary habits and children’s nutritional status. The consumption of whole grains a few times per week (OR = 0.56, 95% CI: 0.33–0.95, *p* = 0.033) and fruits a few times per week (OR = 0.52, 95% CI: 0.32–0.85, *p* = 0.021) were associated with lower odds of high BMI-for-age, suggesting a protective effect on weight status. Conversely, consuming plant-based proteins a few times per week increased the likelihood of higher BMI-for-age (OR = 1.86, 95% CI: 1.12–3.11, *p* = 0.016). Rarely eating or skipping breakfast was associated with lower odds of being in a higher BMI-for-age category (OR = 0.30, 95% CI: 0.14–0.63, *p* = 0.001). For height-for-age, males were more likely to be stunted than females (OR = 1.87, 95% CI: 1.22–2.88, *p* = 0.004). Vegetable consumption a few times per week showed a weak association with stunting (OR = 1.09, 95% CI: 0.10–2.07, *p* = 0.030), while infrequent intake of plant proteins was linked to lower odds of stunting (OR = 0.45, 95% CI: 0.01–0.90, *p* = 0.043). Overall, the consistent consumption of fruits, whole grains, and breakfast appears beneficial for maintaining healthy growth and nutritional status among children.

### 3.1. Differences in Dietary Quality Score Across Income Categories and Marital Status

The one-way ANOVA test revealed a statistically significant association between income and dietary quality score (*p* < 0.001). Children from households with a salary of more than ZAR 10,000 to ZAR 20,000 had a higher mean dietary quality score (mean = 70.86, SD = 10.78) and those from households earning less than ZAR 5000 had the lowest dietary quality score. A statistically significant association was observed between marital status and dietary quality score (*p* < 0.001). Children from households with married couples had higher dietary quality scores, while those from divorced, cohabiting, and widowed households had lower quality scores that are almost similar, ranging from 51.1 to 52.8.

### 3.2. Sex Differences in Dietary Quality Score

A two-sample t-test showed no statistically significant association between sex and dietary quality score (*p* = 0.7094), with both females and males having the mean dietary quality score of 55.

### 3.3. Association of Age, Sex, and Income with Dietary Quality Score

The multiple linear regression analysis showed income as the strongest predictor of better dietary scores while adjusting for age and sex. Children from families earning more than ZAR 20,000 and ZAR 10,000 to ZAR 20,000 were significantly associated with better dietary quality scores compared to those from households earning less than ZAR 5000.

## 4. Discussion

This study examined the influence of socio-demographic factors and dietary habits on the nutritional status of school-aged children. While existing studies have examined links between family background, eating patterns, and child nutrition, this research offers a distinct contribution by focusing on a relatively neglected group—children aged 8 to 12 living in rural South Africa, specifically within the Thulamela Municipality of Limpopo Province. A notable outcome of the study is the identification of both stunting and overweight conditions/obesity among the same group of children, highlighting the double burden of malnutrition, a phenomenon that has received limited focus in this particular population and setting. By assessing a combination of socio-demographic variables (including parental education level, employment, and marital status) and dietary behaviors (such as routine breakfast intake and the consumption of whole grains, dairy products, and vegetables), this study provides a holistic view of the range of influences shaping nutritional outcomes in resource-constrained environments. These findings offer valuable evidence to support the creation of targeted nutrition programs in both schools and communities, adding region-specific insights to the broader body of knowledge.

The findings indicate a dual burden of malnutrition, with stunting occurring alongside overweight conditions and obesity. This coexistence reflects a complex nutritional transition increasingly observed in South Africa [24,25]. Despite national nutrition programs, stunting remains common, particularly among children in rural and low-income households, highlighting ongoing public health challenges [26,27]. The presence of both under- and overnutrition is concerning, as stunted children face impaired growth and cognitive development, while overweight and obese children are at higher risk for non-communicable diseases such as type 2 diabetes, hypertension, and cardiovascular disorders later in life.

The results also demonstrate that higher levels of parental education and household income are associated with improved height-for-age and BMI-for-age growth status. Parents with higher education levels typically have a greater knowledge of nutrition, feeding practices, and health promotion, which supports healthier dietary choices and better growth outcomes in children [28,29]. Likewise, increased household income enables access to a wider range of nutritious foods and better healthcare, reducing the risk of undernutrition and growth-related problems [30,31]. Evidence from other African countries similarly shows that maternal education and economic stability are protective factors against stunting and poor growth [32,33]. In low-income households, food insecurity and a reliance on inexpensive, calorie-dense foods increase the likelihood of both undernutrition and overweight conditions.

Regarding dietary habits, regular breakfast consumption, daily vegetable intake, and consistent dairy consumption were positively associated with growth outcomes. Breakfast provides essential nutrients that support linear growth and healthy body composition, while skipping this meal is linked to lower nutrient intake and suboptimal growth [34]. Vegetables contribute important vitamins, minerals, and dietary fiber necessary for proper growth, and insufficient intake can negatively impact anthropometric measures [35]. Dairy products supply high-quality protein, calcium, and other nutrients crucial for growth, and regular consumption has been associated with higher height-for-age and better BMI-for-age Z-scores in children [36]. Together, these dietary practices promote adequate nutrient intake and support healthy growth, particularly in populations experiencing the double burden of malnutrition [37].

Household income emerged as a key determinant of dietary diversity. Most households had incomes less than ZAR 5000 per month, limiting access to a variety of nutritious foods. Low-income families often rely on staple foods such as maize meals or rice, which provide energy but insufficient vitamins and minerals, resulting in poorer dietary diversity and higher vulnerability to food insecurity [24,26]. Economic constraints may also lead to reliance on low-cost, ultra-processed foods, increasing the risk of both nutrient deficiencies and being overweight.

The findings of this study indicate that the consumption of fruits and whole grains a few times per week was associated with a reduced likelihood of having a high BMI-for-age, suggesting that these food groups may contribute to maintaining a healthy body weight among children. This observation aligns with recent studies showing that higher whole-grain intake is inversely associated with BMI and obesity risk in children [38,39,40]. Similarly, the consumption of fruits a few times a week has been linked to a lower prevalence of overweight conditions and obesity in school-aged children [41,42]. The present study found that children who rarely consumed breakfast were significantly less likely to have a high BMI-for-age compared to those who ate breakfast regularly. This finding suggests an inverse association between breakfast frequency and BMI, which contrasts with much of the existing literature. Typically, regular breakfast consumption is linked to improved weight regulation and a lower risk of overweight conditions or obesity among children [34,43]. Conversely, the intake of plant-based proteins a few times per week was associated with higher BMI-for-age, potentially reflecting energy-dense meals or large portion sizes, although this should be interpreted cautiously as nutrient composition was not assessed [38].

Regarding height-for-age, male participants were more likely to be stunted than their female counterparts, supporting recent findings that boys are more vulnerable to growth faltering in resource-limited settings [40]. The weak association between occasional vegetable consumption and stunting may reflect inadequate dietary diversity rather than a direct effect of vegetable intake. Interestingly, children who rarely consumed plant proteins were less likely to be stunted, which could be explained by a substitution with animal-based proteins that provide higher-quality amino acids essential for growth [39,42]. Overall, these results highlight the importance of diverse and balanced diets, particularly the consistent intake of fruits, whole grains, and breakfast, in promoting healthy growth and preventing malnutrition among children.

### Strengths and Limitations

This study offers valuable insights into the double burden of malnutrition among school-aged children in a rural South African setting, providing a multidimensional understanding of how socio-demographic factors and dietary habits influence nutritional status. Its comprehensive assessment of anthropometric indicators and dietary practices contributes policy-relevant evidence to inform school nutrition programs and community health interventions. However, the cross-sectional design limits causal interpretation of the observed associations, and the reliance on self-reported dietary data may have introduced recall bias. Additionally, the study’s focus on selected villages within Thulamela Municipality restricts generalizability to other settings, while seasonal variations in food availability were not accounted for and may have influenced the reported dietary patterns. The snowball sampling method used to identify eligible children could have introduced selection bias, as participants are likely to refer peers within their own social circles, reducing the diversity and representativeness of the sample. Additionally, the reliance on self-reported dietary information could be subject to social desirability bias, with participants or caregivers potentially overreporting food intake or underreporting unhealthy eating habits to align with perceived expectations.

## 5. Conclusions

The double burden of malnutrition with a high rate of stunting and overweight conditions and obesity in rural villages in Thulamela Municipality is a significant concern. Higher levels of parental education and income correlated positively with better height- and BMI-for-age growth status. Regular breakfast intake, daily vegetable consumption, and dairy intake were associated with better growth indices. Over half of the households earned <ZAR 5000 monthly, limiting dietary diversity. The study revealed that parental education, marital status, and household income were key socio-demographic factors influencing children’s height-for-age and BMI-for-age. This suggests that the socio-economic environment and family circumstances have a significant impact on children’s nutritional status and overall growth.

## 6. Recommendation

Addressing economic disparities, improving dietary diversity, and promoting healthy eating habits are critical to mitigating the double burden of malnutrition and supporting optimal child growth. Nutrition education initiatives should be strengthened in both schools and communities to promote healthy eating behaviors among children and their caregivers. Policies that promote healthy school food environments should be implemented and enforced. These should include providing nutritious meals at schools, limiting the sale of ultra-processed foods, and regulating the marketing of unhealthy food products to children.

## Figures and Tables

**Table 1:** Socio-demographic characteristics of the study participants and their association with nutritional indicators (*n* = 347).

Characteristics	Frequency (*n*)	Percentage (%)	HAZ *p*-Value	BAZ *p*-Value
Sex			0.012	0.348
Girls	184	53.0		
Boys	163	47.0		
Parental education			0.027	0.692
None	17	4.9		
Primary	14	4.0		
High school	149	42.9		
Tertiary	167	48.1		
Parental marital status			0.001	0.034
Cohabiting	35	10.1		
Married	123	35.4		
Single	159	45.8		
Widowed	30	8.6		
Employment status			0.076	0.077
Employed	160	46.1		
Unemployed	187	53.9		

**Table 2:** Household income and expenditure on food and their association with nutritional indicators (*n* = 347).

Characteristics	Frequency (*n*)	Percentage (%)	HAZ *p*-Value	BAZ *p*-Value
Monthly household income			0.008	0.043
<ZAR 5000 (USD28.89)	199	57.3		
ZAR 5000–ZAR 10,000 (USD 288.93–577.86)	66	19.0		
ZAR 10,000–ZAR 20,000 (USD 577.86–1155.72)	44	12.7		
>ZAR 20,000 (USD 1155.72)	38	11.0		
Money spent on food per month			0.343	0.480
<ZAR 2000 (USD 115.57)	199	57.3		
>ZAR 2000 (USD 115.57)	148	42.7		

**Table 3:** Food consumption frequencies and their association with nutritional status.

Food Item	Frequency	(%)	HAZ *p*-Value	BAZ *p*-Value
Whole grains (daily)	20	5.8	<0.001	0.107
Vegetables (daily)	277	79.8	0.012	0.357
Fruits (daily)	74	21.3	0.158	0.371
Dairy (daily)	103	29.8	0.132	<0.001
Sugary drinks (daily)	130	37.5	0.573	0.392
Fast food (a few times/week)	99	28.5	0.149	0.158

**Table 4:** Meal frequencies and their association with nutritional indicators.

Meal	Category	Frequency	%	HAZ *p*-Value	BAZ *p*-Value
Breakfast	Everyday	220	63.4%	<0.001	0.051
Lunch	Everyday	305	87.9%	0.593	0.566
Dinner	Everyday	322	92.8%	0.846	0.153

**Table 5:** Association between dietary habits and nutritional status indicators (BMI-for-age and height-for-age).

Variables	Category	OR	95% CI	*p*-Value
BMI-for-age				
Whole grain	A few times per week	0.56	0.33–0.95	0.033
Fruits	A few times per week	0.52	0.32–0.85	0.021
Plant protein	A few times per week	1.86	1.12–3.11	0.016
Breakfast	Rarely	0.30	0.14–0.63	0.001
Height-for-age				
Sex	Male	1.87	1.22–2.88	0.004
	Female	Ref		
Vegetables	A few times per week	1.09	0.10–2.07	0.030
Plant protein	Rarely	0.45	0.90–0.01	0.043

## Data Availability

The data presented in this study are available on request from the corresponding author. The data are not publicly available due to privacy and ethical reasons.
